# A novel approach to low-cost, rapid and simultaneous colorimetric detection of multiple analytes using 3D printed microfluidic channels

**DOI:** 10.1098/rsos.231168

**Published:** 2024-01-17

**Authors:** Piyush Mishra, Sagar Navariya, Priyanshi Gupta, Bhupendra Pratap Singh, Samridhi Chopra, Swapnil Shrivastava, Ved Varun Agrawal

**Affiliations:** ^1^ CSIR-National Physical Laboratory, Dr. K.S. Krishnan Marg, New Delhi 110012, India; ^2^ Academy of Scientific and Innovative Research (AcSIR), Ghaziabad 201002, India; ^3^ Liquid Crystal Research Laboratory, Department of Physics, University of Lucknow, Lucknow 226007, India; ^4^ Department of Electro-Optical Engineering, National United University, Miao-Li-360, Taiwan

**Keywords:** colorimetric detection, drug detection, DIY RepRap 3D printer, μPADs

## Abstract

This research paper presents an inventive technique to swiftly create microfluidic channels on distinct membrane papers, enabling colorimetric drug detection. Using a modified DIY RepRap 3D printer with a syringe pump, microfluidic channels (µPADs) are crafted on a flexible nylon-based substrate. This allows simultaneous detection of four common drugs with a single reagent. An optimized blend of polydimethylsiloxane (PDMS) dissolved in hexane is used to create hydrophobic channels on various filter papers. The PDMS-hexane mixture infiltrates the paper's pores, forming hydrophobic barriers that confine liquids within the channels. These barriers are cured on the printer's hot plate, controlling channel width and preventing spreading. Capillary action drives fluid along these paths without spreading. This novel approach provides a versatile solution for rapid microfluidic channel creation on membrane papers. The DIY RepRap 3D printer integration offers precise control and faster curing. The PDMS-hexane solution accurately forms hydrophobic barriers, containing liquids within desired channels. The resulting microfluidic system holds potential for portable, cost-effective drug detection and various sensing applications.

## 1. Introduction

Using paper as a substrate material for analytical testing dates back to the early 1800s with the involvement of litmus paper. Low-cost, flexibility and portability, power-free flow of fluids via capillary action, high ratio of surface area to volume for colorimetric detection and the ability to store reagents in active form within a fibre network are some of the unique features of paper that make paper-based sensing more valuable than traditional devices [1]. The pioneering works of George M. Whitesides' group make the paper one of the most emerging technologies for sensing applications. First paper-based microfluidic sensor (microfluidic paper-based analytical device (µPAD)) was presented in 2007 by Martinez *et al*. [2]. Their work presented portable low-cost bioassays on paper substrate µPAD patterned by photolithography [2,3]. Afterwards, multiple procedures for paper µPADs technology evolved [4] such as wax printing [5,6], paper folded mask [7], stamp lithography [8], screen printing [9–11], inkjet printing [12,13], vapour phase deposition [14], laser-toner printing [15,16] and 3D printing [17,18] became typical for designing hydrophobic microfluidic channels on paper. These microfluidic channels provide low-cost, portable and flexible diagnostic platforms. The paper-based microfluid devices can be applied to perform several sensing applications, including enzymatic detection, colorimetric detection, fluorescence detection mechanisms and electrochemistry [19]. Several biosensing applications are performed via paper-based devices including colorimetric detection of phenolic compounds using paper-based bio-assays [20], detection of pathogens, food safety and environmental constituents, immunoassay designing, DNA sensing [19]. Paper-based devices have been use for tuberculosis detection using colorimetric gold nanoparticles [21]. Chiang *et al*. mentioned 3D wax-printed paper-based microfluidic barriers for glucose and nitrite assays in 2018 [6]. Polyurethane acrylate (PUA)-based low-cost microfluidic channel fabrication techniques discussed in the work of Lin *et al*. is yet another method for µPADs patterning [22,23]. Whatman grade 1 filter paper is used as substrate and microfluidic channels are patterned using water-based PUA. The designed patterns were masked on PUA paper and exposed to UV light [22]. Nitrocellulose membrane papers were patterned with the same techniques for designing enzyme-linked paper-based immunosorbent assays [23]. This method is, however, based on conventional lithographical techniques. Mask-based lithographical procedures have their limits as a mask designed can be used only for a single pattern of µPADs, and by change of design of µPADs, the masks are required to be changed. The field of 3D printed microfluidic channels is rapidly advancing, offering significant benefits and enhanced user-friendliness. Puneeth *et al*. conducted a professional study that focused on the optimization and development of 3D printed microfluidic paper-based analytical devices (µPADs) for sensing applications. Their research used an image processing mechanism to accurately measure the viscosity of biological samples [24,25]. The study mentioned by Zargaryan *et al*. [17] examines the utilization of polypropylene filament for designing 3D printed paper microfluidic channels. These channels are subsequently cured for a minimum duration of 45 min at 175°C. While the channels demonstrate efficiency for point-of-care applications, the design process entails complexity, and the required curing time is relatively high. Modified 3D printers have also found application in the printing of hydrophobic barriers on various types of paper, utilizing wax and polycaprolactone (PCL) printing techniques. However, this approach presents certain drawbacks, primarily in the form of intricate printer modifications and extended curing time [26]. Different curing temperatures significantly influence micro-channel width, and the curing time is dependent on curing temperature [27]. Also the viscosity of polydimethylsiloxane (PDMS) and hexane solution plays vital role on spread and width of patterned barriers [28]. Paper-based µPADs are used for colorimetric sensing, paper chromatography spot metal detection, microfluidic applications, and lateral flow immunoassays. In our work, some famous and commonly used drugs are applied on the 3D printed paper µPADs, and their colorimetric test is done on paper to demonstrate their presence and to prove the effectiveness of printed microfluidic channels. The hazards and new issues associated with drug usage include tampering with traditional drugs [29]. Illegal medications have always included other ingredients besides the purported active component, posing a risk to one's health or perhaps causing early death. To intensify the action of drugs, adulterants are being added deliberately in dilute form, or bulk amounts [30]. Paracetamol and aspirin come under pharmacologically active drugs, which can produce adverse health conditions when taken in bulk. A drug like salicylic acid is used in milk as a preservative to enhance the valuable life period [31]. The consequences are knowingly ignored, including asthma and gastrointestinal, kidney and liver problems. Paracetamol is a traditional drug used to treat high temperatures and pain. It is such a classical drug that most of the research that includes the hazardous effects was not conducted until [32]. Drug usage is linked with kidney malfunctioning, bleeding in the stomach and increased mortality rate. Ciprofloxacin, a quinolone antibiotic, is used to cure infections caused by microorganisms, mainly bacteria and it is prescribed for several conditions including thyroid fever, urinary tract infections, skin infections, respiratory tract infections and infectious diarrhoea [16,33]. Aspirin is a proven drug found to be very effective for treatment related to blood thinning, arthritis, muscle ache and minor pain. A low dose of aspirin for long-term treatment of heart attacks is also recommended [34,35]. One of the most common and popularly known analgesic and antipyretic drugs is paracetamol. Although the use of paracetamol is associated with a few side effects, including liver intoxication and gastrointestinal disorders, paracetamol has found its place as one of the most commonly used drugs [36]. Salicylic acid, a keratolytic agent, is a key ingredient for skin care products and is used for medications related to skin disorders [37]. Although these drugs are commonly used in medications, they share the same reagent, ferric chloride (FeCl_3_), for colorimetric detection. This aberrant feature of these drugs makes them eligible for colorimetric detection on printed µPADs. There are numerous techniques and methods to detect these drug impurities in a sample such as spectroscopic methods [38,39], immunoassays [40,41] and gas chromatography–mass spectrometry (GC-MS) [42–44]. Sulfur-specific impurities detection in cimetidine drug using mass spectrometry was discussed by Evans *et al*. [39]. Recommendations for the optimization and design of immunoassays used for drug detection were presented in the work of Mire-Sluis *et al*. [41]. A review of the development and applications of heterogeneous and homogeneous immunoassays for drug detection was presented by Dinis-Oliveria [40]. Control and identification of impurities for the development of drug-detection substances using the GC-MS procedure was discussed in the study published by Lee *et al*. [42]. Impurity *N*-nitroso dimethylamine in valsartan drug substances using the same technique was analysed by Tsutsumi *et al*. [43]. These techniques include expensive equipment and time taking processes.

Table 1 presents a comprehensive comparison of fabrication techniques employed in the production of traditional µPADs. Additionally, this table also highlights certain limitations associated with the procedures discussed in the present study.

**Table 1.** Comparison of traditional techniques of µPADS fabrication.

process of µPADs fabrication	equipment	materials	limitations	references
wax printing	wax printers	wax	mask preparation, uncontrolled flow	Carrilho *et al*. [5]
screen printing	customized masks	wax, hydrophobic materials	mask preparation, requires wax protocol, heating required, printer not available,	Sameenoi *et al*. [9]; Dungchai *et al*. [45]
lithography	UV lamp	photoresist, hydrophobic material	paper modification, UV exposure, one designing pattern at a time	Lin *et al*. [22,23]
paper folding and stamps	customized masks	wax, hydrophobic material	masks preparation, one designing pattern at a time	Xie *et al*. [7]
inkjet printing	inkjet printers	customized liquid ink	ink printing, wax printing, one pattern at a time	Apilux *et al*. [12]; Yamada *et al*. [13]
vapour phase deposition	customized reaction chamber	multiple chemicals	complex technique	Haller *et al.* [14]
laser toner printing	toner laser printer	customized toner, dye	complex technique, requires uniform heating procedures	Ng *et al*. [15]
3D printing	3D printer, filament material	polypropylene, wax, polycaprolactone (PCL)	complex procedure, high curing time	Zargaryan *et al*. [17]; Fu & Wentland [26]
3D printer assembled with syringe pump	3D printer, syringe pump	PDMS, hexane	requires optimization of the flow rate, high standard deviation of barrier width, viscosity and printing speed	present work

This study presents an innovative approach to enhance the fabrication of µPADs with a focus on cost-effectiveness. Specifically, the method explores the detection of commonly used drugs using these devices. To achieve this, a modified DIY RepRap 3D printer, integrated with a syringe pump, is employed to generate hydrophobic patterns on hydrophilic membranes. Compared with previous techniques involving modified desktop *x*-*y* plotters [28], this approach offers enhanced control and simplifies the design process. By using a 3D printer with *z*-axis motion, multiple barriers are simultaneously plotted, significantly reducing the required curing time. This technological advancement enhances overall efficiency and enables the production of barriers with greater ease. Furthermore, the printer's hot plate, capable of reaching temperatures up to 110°C, facilitates instant curing of PDMS on paper, eliminating the need for lithography and mask patterning. Consequently, multiple channels can be formed on any paper substrate. The printer also offers customization options, enabling the simultaneous patterning of various types of µPADs. PDMS, a low-cost chemical that cures into an elastomeric material, provides several advantages. The channels on the paper can be flexed or folded without compromising their integrity. The paper membranes, along with different flow rates of PDMS and hexane solutions, are compared to optimize the channel width at consistent curing temperatures and times. The paper-based microfluidic channels demonstrate colorimetric detection of commonly used drugs using a single detector reagent (FeCl_3_).

## 2. Experimental set-up

### 2.1. Materials and reagent

Filter papers of distinct types are used for designing patterns, including Whatman grade 1, Whatman grade 41, Immunodyne ABC, Biodyne C and Biotrace NT of pore sizes 11, 20, 0.45, 0.45 and 0.2 µm, respectively. The filter papers Whatman grade 41 and Whatman grade 1 have cellulose membranes, Biotrace NT have nitrocellulose membrane, Immunodyne ABC and Biodyne C have Nylon 6,6 membranes.

Silicon elastomer (PDMS precursor) is procured from Dow Corning, namely Sylgard 184 dual component suit. Hexane is procured from Fluka chemical. Ciprofloxacin hydrochloride (LR, CAS-No. 86393-32-0, purity 98%), aspirin (LR, CAS-No. 50-78-2, purity 99%), paracetamol (LR, CAS-No. 103-90-2, purity 99%) and NaOH (AR, CAS-No.1310-73-2, purity 99.5%) are supplied by CDH fine chemicals (Central Drug House (P) Ltd) and used without any further modifications. Salicylic acid (ACS, CAS-No. 69-72-1, purity 99%) is supplied from Fisher Scientific. Ferric chloride (CAS-No.7705-08-0 purity 98%) is obtained from Merck-Schuchardt. H_2_SO_4_ (AR, CAS-No. 7664-93-9, purity greater than 98%) is supplied from High Purity Laboratory Chemicals Pvt Ltd (HPLC) and acetone (ER, CAS-No. 67-64-1, purity 99.5%) is procured from Thermo Fisher Scientific India Pvt Ltd.

### 2.2. Methodology

**Figure 1.**
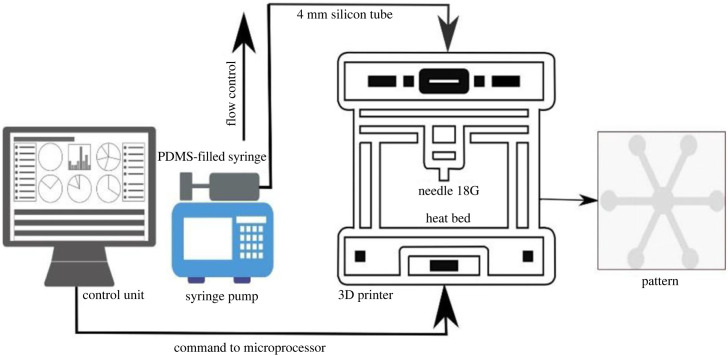
Schematic of the experimental set-up.

DIY RepRap 3D printer is assembled using Arduino AtMega 2560 board and open-source firmware marlin 2.0.7. The hot bed ‘MK2B’ is used as substrate below the platform to ensure uniform heating and is set to reach a maximum temperature of 110°C. An inductive proximity sensor assembled to the printer's extruder facilitates the bed levelling process and acts as a *z*-axis minimum end stop switch. The fluid infusion is regulated using a syringe pump, make ‘Harvard Apparatus PHD 2000 Programmable’. PDMS is mixed with hexane in a ratio of 1 : 2 to obtain the required viscosity. The solution is then filled into a 50 ml syringe attached to a syringe pump. The flow rate is optimized to 3 µl min^−1^, and the motion of the printer is set at 1000 mm min^−1^ for continuous dispensing of the solution for patterning. The PDMS mixture is dispensed on paper using 18-gauge syringe needle connected to extruder via 4 mm silicon tube from the syringe. For maintaining uniform flow, the needle is tapered. Patterns are designed in ‘Inkscape 0.92.5’ open-source software and are converted to G-codes using the G-code extension of Inkscape. ‘Pronterface 2017’ software is used for interfacing the 3D printer with/to the computer. A schematic of the fabrication set-up is shown in figure 1. (Initial configuration of the set-up is shown in electronic supplementary material, figure S1.)

The contact angle measurements are carried out to determine the wettability of the filter papers and to assess hydrophobicity of printed channels for use in µPADs using the Dataphysics OCA 15 EC equipment.

In addition, the topography of filter papers with small pore size compared with Whatman filter papers is analysed using atomic force microscopy (AFM). AFM analysis is conducted on both uncoated and PDMS-coated filter papers using the Multimode 8 Bruker system using tapping mode. This system features high-resolution imaging of surfaces, and the J Piezoelectric scanner used in the analysis is capable of measuring a scanning area of 120 × 120 µm with a vertical range of approximately 5 µm. The AFM system is interfaced with NanoScope analysis software, and the radius of the tip used for AFM analysis of samples is 8 nm.

These techniques are employed to provide a thorough understanding of the surface properties and hydrophobicity of the filter papers and their suitability for use in printed µPADs. The results of the contact angle measurements and AFM analysis will help determine the effectiveness of the filter papers in microfluidic applications and aid in the development of improved µPADs.

### 2.3. Preliminary solution preparation

The solutions for aspirin, salicylic acid, paracetamol, ciprofloxacin, and the reagent FeCl_3_ are prepared according to the described procedure.

1. **Aspirin.** A solution containing 5 mg of aspirin is dissolved in 20 ml of deionized water (DI) and subjected to continuous stirring at a temperature of 80°C. Subsequently, a slow addition of a 0.1 M NaOH solution is introduced into the solution. This process is sustained for a duration of 25 min. To initiate the hydrolysis of aspirin, a small quantity of dilute sulfuric acid is added to 10 ml of this solution.
2. **Salicylic acid.** To prepare a solution, 0.5 mg of salicylic acid is dissolved in 1 ml of acetone solution. Then 19 ml of DI is added to the mixture.
3. **Paracetamol.** 40 mg paracetamol is dissolved in 10 ml DI to make clear solution.
4. **Ciprofloxacin.** 10 mg of ciprofloxacin is dissolved in 60 µl acetic acid along with 10 ml of DI.
5. **Ferric Chloride.** To prepare ferric chloride (FeCl_3_) reagent solution, 50 mg of FeCl_3_ is dissolved in 30 ml of DI. This ensures clear observation of colorimetric changes during the reaction with the specified drugs.

The tabulated representation of the preliminary solution preparation procedure can be found in electronic supplementary material, table S1.

## 3. Results and discussion

### 3.1. Optimization of designing patterns

**Figure 2.**
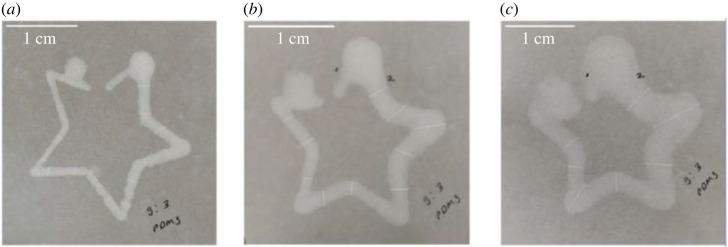
Width analysis on Whatman grade 41 filter papers after (*a*) 1 h, (*b*) 3 h and (*c*) 20 days after plotting.

Filter papers of different types and with distinct pore sizes are studied for designing PDMS patterns. Figure 2 shows the spread of PDMS patterns with time on Whatman grade 41 paper.

From figure 2, it is evident that the width of the designed PDMS polygon spreads with time on Whatman grade 41 filter paper having a pore size of 20 µm. The same behaviour is also observed on Whatman grade 1 filter paper of pore size 11 µm. The spread of patterns over time is influenced by multiple factors, including the pore size and surface roughness of the filter papers. The spreading of barriers can modify the available area, specifically the channels, on the filter paper, thereby impacting the performance of the µPADs. Consequently, it is crucial to carefully consider the properties and characteristics of the filter papers during pattern design to ensure the integrity of pattern dimensions. We observed that the filter papers with pore size equal to or smaller than 0.45 µm are highly resistant to the spreading of the designed patterns.

**Figure 3.**
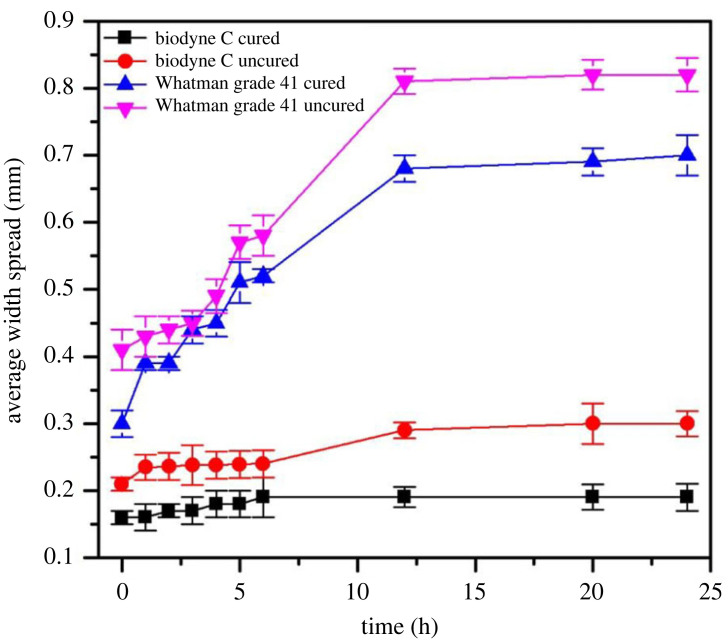
Spreading of designed patterns width with respect to time.

To study the spread of designed patterns, the widths are measured at regular interval, three 3 cm lines were printed on pairs of Biodyne C and Whatman grade 41 papers. One of each paper type underwent a curing process for 9 s at 150°C, while the other remained uncured. Imaging was performed at specific time intervals, including every hour for 6 h, as well as at 12 and 24 h post-printing. The acquired images were subjected to quantitative analysis using ImageJ, and 30 measurements were obtained for each of the printed lines to assess width variations on the different paper substrates. The average width measurements for all three lines along with the standard deviation are plotted over time for all cases, as shown in figure 3. The findings clearly indicate that Whatman grade 41 filter papers exhibit wider width profiles and a higher rate of width spreading compared with Biodyne C papers. Furthermore, the curing process proved to be essential in preventing the undesirable spread of line widths.

**Figure 4.**
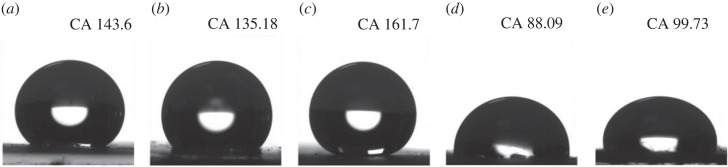
Contact angle (CA) measurements of printed µPADs on (*a*) Biodyne C, (*b*) Biotrace NT, (*c*) Immunodyne ABC, (*d*) Whatman grade 41 and (*e*) Whatman grade 1 filter papers.

Figure 4 presents the contact angle measurements conducted on the printed region of the µPADs to assess their hydrophobicity. It is noteworthy that the water drop remained stable on Biodyne C, Biotrace NT and Immunodyne ABC membranes for several minutes, while on Whatman grade filter paper, it is quickly absorbed within seconds. The images captured immediately after the drop placement highlight these observations.

**Figure 5.**
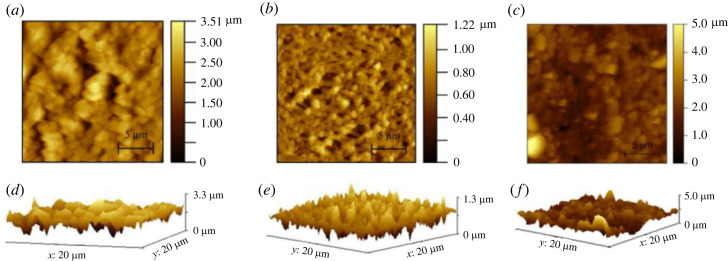
AFM images of (*a*) Biodyne C, (*b*) Biotrace NT and (*c*) Immunodyne ABC without PDMS coating. (*d*–*f*) represent AFM-obtained three-dimensional view of roughness of the filter papers, respectively.

Electronic supplementary material, table S2 provides a comprehensive comparison of different properties of the filter papers. To analyse the topography and roughness of filter papers, AFM is performed on Biodyne C, Biotrace NT and Immunodyne ABC with and without the application of PDMS. Each paper is scanned for 20 × 20 µm under tapping mode. The mean roughness of the papers before and after treatment with PDMS is measured and observed to be 395.1 and 271.7 nm for Biodyne C, 150.8 and 185.2 nm for Biotrace NT, and 1014 and 391.9 nm for Immunodyne ABC, respectively. Surface roughness plays a crucial role in determining the wettability of papers as it affects the wetting of the surface [46,47]. Papers typically have a fibre-like structure resulting in the rough surface, forming capillary and resulting in hydrophilic behaviour. However, decreasing roughness has an adverse effect on fluid spread, resulting in hydrophobicity. Therefore, the application of PDMS on filter papers leads to a decrease in roughness and an increase in hydrophobicity. Figure 5 shows the AFM images of Biodyne C, Biotrace NT and Immunodyne ABC without the application of PDMS.

**Figure 6.**
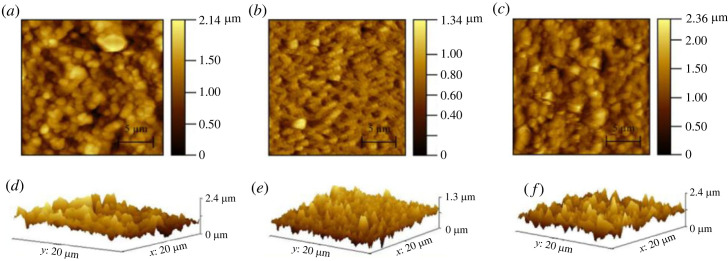
Atomic FM images of (*a*) Biodyne C, (*b*) Biotrace NT and (*c*) Immunodyne ABC with PDMS coating. (*d*–*f*) represent AFM-obtained three-dimensional view of roughness of the filter papers, respectively.

AFM images of these papers after application of PDMS are shown in figure 6.

**Figure 7.**
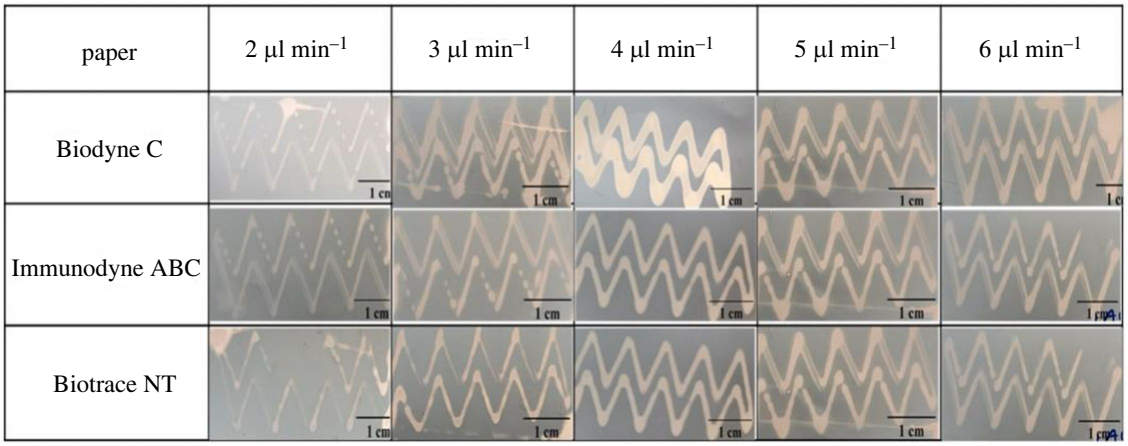
Analysis of different infusion rates of PDMS-hexane solution on different filter papers.

The surface properties obtained from AFM findings validate the contact angle measurements, as the implementation of PDMS effectively diminishes the roughness of the papers. As a result, the hydrophobic properties are enhanced, leading to significantly higher contact angle values compared with papers that have not been treated with PDMS. The infusion rate of the syringe pump plays a crucial role in achieving precise patterning of the PDMS-hexane solution on filter papers. A higher infusion rate can result in uncontrolled spreading of the solution, leading to non-uniform patterns. Conversely, a lower infusion rate may supply an insufficient quantity of solution, resulting in incomplete and discontinuous patterns. To address these challenges and achieve consistent patterns, a study is conducted on three different filter papers, as illustrated in figure 7, to investigate pattern design at various infusion rates.

**Figure 8.**
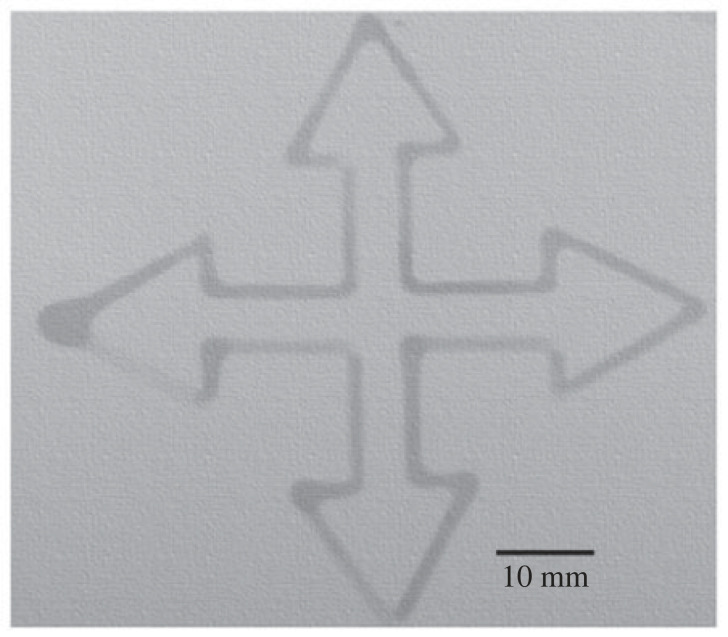
Microfluidic channels on Biodyne C filter paper.

The findings reveal that an infusion rate of 5 µl min^−1^ is highly suitable for a solution ratio of 1 : 2, providing a continuous flow for patterning designs on filter papers with minimal thickness and minimal spread. The study examined infusion rates ranging from 2 to 6 µl min^−1^ to determine the optimal infusion rate for the desired outcomes. Nylon membranes have high mechanical strength in comparison with nitrocellulose membranes. This is due to the long strands and orderly hydrogen bonds in nylon. Additionally, these membranes are re-usable as their pores remain intact after washing [48,49]. In the present work, capillary action is used for the primary transfer technique, taking advantage of this property. Considering the benefits of using nylon membranes for colorimetric detection in printed microfluidic channels, Biodyne-C paper substrate, which also has a nylon 6,6 base material and 0.45 µm pore size, is selected over Immunodyne ABC paper. This decision is made considering the lower roughness parameter of Biodyne-C paper. Microfluidic patterns for colorimetric sensing of different drugs are designed on this filter paper, as shown in figure 8.

The optimization of barrier width is performed using the ImageJ open-source software. The calibration of the image size is accomplished employing a ruler with 0.5 mm markings. For figure 8, a total of 150 measurements of line width distances are taken to determine the lengths of the barriers. The average width of the barriers is found to be 1.783 mm, with a standard deviation of 0.698.

The curing process for PDMS-hexane solutions involves two stages. Firstly, the solutions are cured at 110°C on a heated bed during the printing process. Subsequently, the printed designs are immediately transferred to a preheated muffle furnace set at 150°C for a duration of 10 s. This two-stage curing method ensures that the printed design remains intact and prevents any unwanted spreading [27].

### 3.2. Colorimetric analysis of the drugs

#### 3.2.1. Procedure of control experiment

**Figure 9.**
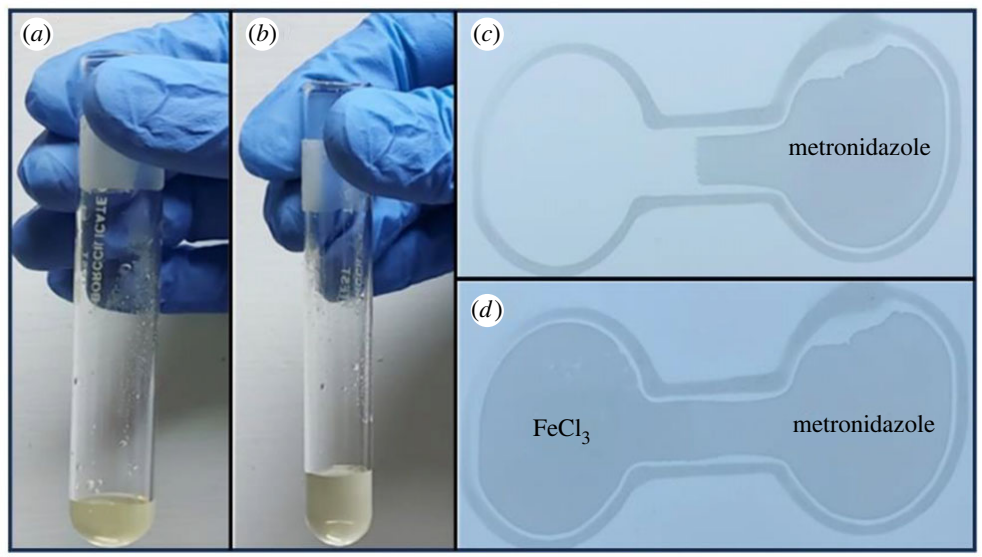
Control experiment showing (*a*) FeCl_3_ (*b*) reaction of metronidazole and FeCl_3_ in test tubes (*c*) metronidazole on µPADs (*d*) reaction of metronidazole and FeCl_3_ on µPADs.

In order to conduct a control experiment aimed at discerning distinct colorimetric reactions of pharmaceutical compounds, an investigation was carried out involving FeCl_3_ and metronidazole. Metronidazole, a frequently prescribed antibiotic–antiprotozoal medication employed for the treatment of bacterial and parasitic infections, features a molecular structure characterized by an imidazole ring, a nitro group (NO_2_), a hydroxyl group (OH) and a methyl group (CH_3_) [50]. Notably, the chemical constituents of metronidazole are devoid of the ability to form colorimetric complexes with FeCl_3_. Consequently, 40 mg metronidazole in 10 ml DI was chosen as the agent for executing the control experiment, ensuring the reliable differentiation of colorimetric responses. The control experiment outcomes are presented in figure 9.

In figure 9*a*, a test tube contains a 1 ml solution of FeCl_3_. Upon introducing 1 ml metronidazole to the test tube, as shown in figure 9*b*, no discernible colour change is observed. In the context of µPADs, the procedure involves initially applying a 5 µl drop of metronidazole to one side, allowing it to fully disperse into the microfluidic channel, as presented in figure 9*c*. Subsequently, 5 µl FeCl_3_ is introduced to the opposite side of the channel, as shown in figure 9*d*. Despite the contact of these two chemicals within the channel, there is no evident colorimetric reaction discernible under these experimental conditions.

#### 3.2.2. Chemical reaction of paracetamol with FeCl_3_

Paracetamol, also known as acetaminophen, undergoes a chemical reaction with FeCl_3_ to yield a 2 : 1 metal–ligand complex with a blue coloration. This reaction is facilitated by the presence of an active hydroxyl group site in the paracetamol molecule, which readily interacts with Fe (III) ions. Specifically, two paracetamol molecules bind to each ferric ion site, while the remaining coordination sites are occupied by water molecules. This process results in the formation of a 2 : 1 complex between paracetamol and FeCl_3_, which is responsible for the blue colour observed in the reaction mixture [51].

#### 3.2.3. Chemical reaction of salicylic acid with FeCl_3_

Most transition metal coordination complexes exhibit colour due to electronic transitions of d-orbital electrons. Salicylic acid is a compound that contains two active protons, one in the phenolic group and another in the carboxylic group, both of which can be readily removed. The phenoxide and carboxylate ion, also known as the salicylate ion, that are formed as a result can easily access the ferric ion present in the solution. This interaction leads to the formation of a violet-coloured complex [52].

#### 3.2.4. Chemical reaction of aspirin with FeCl_3_

On the other hand, aspirin also contains a salicylate ion, but it is not present in a free form and cannot react directly with ferric ion. To activate the salicylate ion in aspirin, hydrolysis must be performed, which removes the acetyl group from the molecule (as shown in electronic supplementary material, figure S4, scheme 3: hydrolysis of aspirin). The resulting salicylate ion can then undergo the same reaction as salicylic acid and react with ferric chloride to form an observable violet colour complex [51].

#### 3.2.5. Chemical reaction of ciprofloxacin with FeCl_3_

Ciprofloxacin's 3 : 1 coordination complex with ferric chloride leads to the formation of a reddish-orange colour in the reaction medium. This occurs due to the quinolone's carbonyl and carboxylic groups, which are nucleophilic and can readily attack ferric ions, resulting in the formation of the coloured complex [53].

The schematic representations of the chemical reactions between each drug and FeCl_3_ can be found in the electronic supplementary material, information, §1.

**Figure 10.**
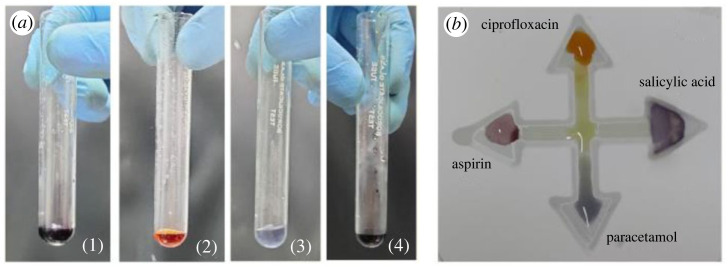
(*a*) Colour indication of different drugs ((1) aspirin, (2) ciprofloxacin, (3) paracetamol and (4) salicylic acid) using a single detector reagent (FeCl_3_) in the test tubes. (*b*) Colour indication of the same drugs on the patterned µPADs.

Colorimetric detection of aspirin, ciprofloxacin, paracetamol and salicylic acid using single reagent FeCl_3_ in test tubes and on fabricated µPADs is shown in figure 10. A precise volume of 10 µl of each drug solution is placed on the designated arms of the µPADs, as shown in figure 8. Following this, a droplet of FeCl_3_ solution is accurately dispensed at the centre of the µPADs. Using the principle of capillary action, the solutions effectively interact and undergo the intended reactions within the microfluidic channels.

In the electronic supplementary material, §5, we present a reverse reaction mechanism in which FeCl_3_ is initially applied to the µPADs. Subsequently, after the complete absorption of FeCl_3_ on the µPADs, drugs are applied to observe colorimetric changes. This is done to demonstrate the feasibility of the reversed reaction procedure.

### 3.3. Estimating the minimum concentration for naked-eye observation

The standard procedure for determining the minimum concentration of drugs observable through the naked eyes involves dispensing 2 µl volume of each drug solution on the designated arms of the µPADs shown in figure 8. Following this, a droplet of FeCl_3_ solution is accurately dispensed at the centre of the µPADs. Using the principle of capillary action, the solutions effectively interact and undergo the intended reactions within the microfluidic channels. In the case of the test tube medium, a mixture comprising 1 ml of FeCl_3_ and 1 ml of each respective drug is used. A comprehensive description of this procedure is presented in the subsequent subsections below.

#### 3.3.1. Paracetamol

Initially, 100 mg of the compound was dissolved in a 10 ml aqueous medium. A drop of 2 µl drug is placed on the paper µPADs, and when the drop is completely absorbed, a 2 µl drop of the FeCl_3_ is applied to observe colour change through. Upon reaction with FeCl_3_, a visible colour change was observed. Subsequent stages of the experiment involved a gradual reduction in the drug concentration, with measurements taken at 95, 90, 85, 80, 75, 70, 65, 60 and 55 mg in 10 ml aqueous solution, respectively. Continuing with the experimental protocol, additional assessments were conducted at 56, 58 and 60 mg of drug dose. Notably, the reliable and distinct alteration in colour, which serves as a clear indicator of minimum concentration, was consistently observed when the concentration level reached 60 mg. Below this threshold, there is an absence of any discernible colour change. Applying the same procedure in test tube medium yields the lowest naked eye observation value of paracetamol 40 mg.

#### 3.3.2. Ciprofloxacin

The experimental process for ciprofloxacin commenced with the analysis of a 5 mg solution of the drug in 10 ml of DI, with no discernible red-yellow coloration observed. The drug quantity in the solution was systematically increased to 7, 9, 11 and 13 mg. It was at the concentration of 13 mg that a reddish-yellow hue became perceptible to the naked eye. Subsequently, the determination of the lowest concentration for naked eye observation was repeated within a narrow range of concentrations, specifically at 11.5, 12 and 12.5 mg. It was at the 12 mg level that the desired coloration, as previously observed, became distinguishable. It is important to note that in the test tube medium, the minimum concentration detectable through naked eyes was ultimately determined to be 10 mg.

#### 3.3.3. Salicylic acid

In the investigation involving salicylic acid, the initial phase commenced with the analysis of a 10 mg quantity of the compound, which was dissolved in a mixture consisting of 1 ml of acetone and 19 ml of DI. Subsequently, the resulting solution underwent a reaction with FeCl_3_, leading to the manifestation of a deep purple hue. This experimental procedure was then replicated with varying amounts of the drug, specifically 8, 6, 4, 2, 1, 0.5 and 0.4 mg, in solution of 1 ml acetone and 19 ml DI. Measurements revealed that the distinct purple coloration ceased to be perceptible at the 0.4 mg level. Further repetitions at the 0.45 mg concentration failed to yield a discernible colour change visible to the unaided eye. Consequently, the lowest observable concentration was established at a concentration of 0.5 mg of the drug, both on paper and within the test tube medium.

#### 3.3.4. Aspirin

For aspirin, the primary challenge centred around the issue of hydrolysis. For each analysis iteration, hydrolysis was systematically performed. The experimentation was initiated with 2, 4, 6 and 8 mg of the drug in 10 ml DI. The lower initial quantities were employed due to the prior determination that salicylic acid exhibited the desired coloration at 0.5 mg in 10 ml DI. At the 8 mg level solution, a clearly distinguishable purple coloration was observed. Further experiments were conducted with 6.5, 7 and 7.5 mg of the drug in 10 ml DI solution, revealing that at the 7 mg level, a distinct and discernible colour change was consistently observed. In test tube medium, the lowest naked eye differentiable concentration is determined at 5 mg concentration.

The values of minimum concentration in mg for each drug is then converted to molarity using equation (3.1)

3.1 $$Molarity=\left\lfloor \frac{W\times 1000}{\mathrm{Molar}\;\mathrm{mass}\times V}\right\rfloor M,$$

where *W* is the weight of samples in grams and *V* is volume in ml.

The value of *V* is 0.002 ml (2 µl) on µPAD and 1 ml in test tube.

The molar mass (g mol^−1^) of ciprofloxacin, paracetamol, salicylic acid and aspirin are 331.346, 151.163, 138.121, 180.158, respectively.

The experimentally optimized values of *W* (in grams) for ciprofloxacin, paracetamol, salicylic acid and aspirin, as recorded for limit of detection (LOD) on µPADs, are $2.38\times 10^{-6},\;1.2\times 10^{-5},\;0.05\times 10^{-6}$ and 2 × 10^−7^, respectively. Conversely, when conducted in test tubes, the corresponding values of *W* (in grams) for these drugs are $0.994\times 10^{-3},\;4\times 10^{-3},\;2.5\times 10^{-5}$ and 0.5 × 10^−3^, respectively.

It is worth highlighting that each experiment was repeated four times to guarantee the precision and consistency of drug quantities and their reactions with FeCl_3_.

Minimum concentration for naked eye detection of paracetamol, ciprofloxacin, aspirin and salicylic acid through naked eyes is listed in table 2.

**Table 2.** Concentration of different drugs for naked eyes in test tube and on paper.

drug	concentration in test tube (M)	concentration on µPADs (M)
ciprofloxacin	0.0029	0.0035
salicylic acid	0.018	0.000181
paracetamol	0.026	0.039
aspirin	0.0027	0.00055

### 3.4. Red, green and blue intensities on printed microfluidic paper-based analytical devices

**Figure 11.**
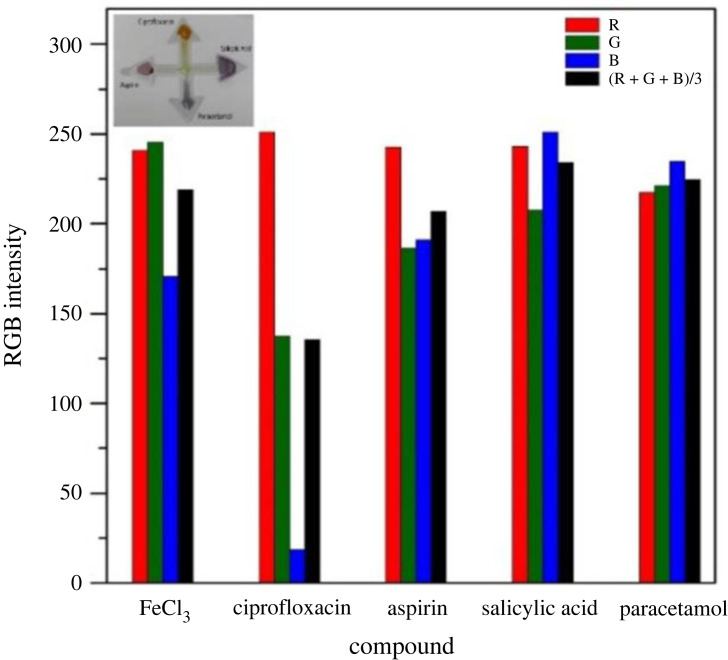
RGB intensities of chromogenic complexes formed by various drugs upon reaction with FeCl_3_ on µPADs.

The red, green and blue (RGB) values corresponding to the colorimetric response of the aforementioned drugs, in conjunction with the reagent FeCl_3_, are acquired through the utilization of the open-source software, ImageJ. The response image is captured and subsequently processed within the ImageJ platform. For each drug, multiple circular (3.7 mm^2^) regions of consistent diameter are chosen, and the average RGB concentration is computed, as shown in figure 11. These RGB values provide a fundamental insight into the attained colour concentration for each drug in conjunction with the reagent. FeCl_3_, when applied to the printed µPADs, exhibits a pale-yellow colour. Upon interaction of ferric chloride with ciprofloxacin, a reddish-yellow colour complex is formed. When hydrolysed aspirin and salicylic acid react with FeCl_3_, they yield a violet colour complex, while the reaction of paracetamol with FeCl_3_ results in the observation of a blue colour on the paper.

### 3.5. Limit of detection measurements

The ultraviolet–visible (UV–VIS) spectroscopic results pertaining to salicylic acid, aspirin, ciprofloxacin and paracetamol have been provided in electronic supplementary material, figure S5. The values of specific wavelength of aforementioned drugs are subjected to validation in accordance with the referenced sources [54–58].

**Figure 12.**
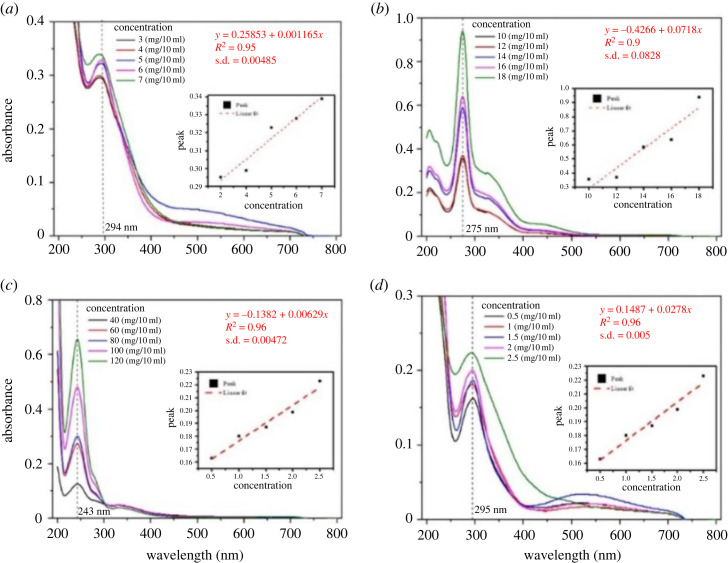
Absorption analysis of: (*a*) aspirin, (*b*) ciprofloxacin, (*c*) paracetamol and (*d*) salicylic acid, along with the corresponding drug concentrations and curve fitting results.

The LOD for the drugs within a test tube medium is determined by the analysis and computational assessment of the standard deviation (s.d.). The interpretation of s.d. involves the utilization of the residual sum of squares (RSS) and degree of freedom (d.f.), as per equation (3.2) [59]. The values of RSS and d.f. are obtained through the curve fitting of the UV absorption spectra of the drugs (figure 12).

3.2 $$s.d.=\frac{\sqrt{\mathrm{RSS}}}{d.f.}$$

The LOD is then obtained through the standard deviation using equation (3.3) [60]

3.3 LOD=3×s.d.

Table 3 lists the values of RSS, d.f., s.d. and LOD.

**Table 3.** Limit of detection of different drugs.

drug	residual sum of squares (RSS)	degree of freedom	standard deviation (mg/10 ml)	limit of detection (mg/10 ml)
paracetamol	0.0067	3	0.00472	0.01416
ciprofloxacin	0.0206	3	0.0828	0.2484
aspirin	7.7083 × 10^−5^	3	0.00485	0.01455
salicylic acid	7.51 × 10^−5^	3	0.005	0.015

Figure 12 shows the plots of absorption spectra (absorbance versus wavelength) of each drug along with the concentrations and linear curve fitting plot. To analyse the dependency of absorption peak value to the concentration of the drugs, five different concentrations of each drug are prepared in accordance with the procedures detailed in §2.3. Through the obtained absorption peak values, linear curve fitting is done to compute the RSS value for estimation of s.d. and LOD. The linear curve fitting equation, *R*^2^ values and standard deviations are also provided in figure 12.

The method presented in this research introduces a novel and advantageous approach that surpasses traditional techniques listed in table 1. It eliminates the need for a clean-room environment, expensive chemicals and photoresists typically used in photolithography methods. Instead, the study employs µPADs, which offer the benefits of being cost-effective and biodegradable. Additionally, the flexibility of µPADs offers a distinct advantage in colorimetric detection and point-of-care applications. It allows for easier integration into various diagnostic devices, offering adaptability to complex geometries and enhanced usability. This flexibility enables µPADs to conform to diverse sample handling requirements, such as folding or bending to facilitate fluid flow, which can be particularly valuable in resource-limited settings and for creating user-friendly, portable and cost-effective diagnostic tools. The DIY printer employed is an open-source RepRap model, enabling individuals to assemble it at a low cost compared with commercial 3D printers. It boasts user-friendliness and delivers faster and responsive outcomes when compared with complex traditional processes like screen printing, photolithography and inkjet printing for µPAD fabrication.

Moreover, the modified 3D printer, integrated with a syringe pump, enables the patterning of microfluidic channels using various materials, including PDMS, modified inks and other hydrophobic fluids. PDMS, being a low-cost material, is particularly suitable for this technique, as the PDMS-hexane solution can be printed and cured simultaneously at the required temperature on the printer's hotbed. Additionally, other affordable chemicals such as polyisobutylene, polystyrene and polypropylene can be used for specific applications. The versatility of the method expands the possibilities for microfluidic channel patterning.

Paper-based microfluidic sensors find applications in various sensing areas, including colorimetric detection of medicinal drugs, detection of heavy metals, analysis of biological fluids, pH detection and concentration-based sensing such as glucose and urea concentration detection. These devices demonstrate a wide range of sensing capabilities and hold promise for further advancements.

## 4. Conclusion

In conclusion, this research demonstrates the successful integration of PDMS and paper using a modified, low-cost, self-designed RepRap 3D printer assembled with a syringe pump. The method offers notable advantages in terms of speed, reliability and user-friendliness, while using affordable and practical materials. By applying a PDMS-hexane solution on various filter papers using the modified 3D printer for µPADs patterning, we have achieved successful colorimetric sensing of aspirin, salicylic acid, paracetamol and ciprofloxacin on the µPADs. These µPADs enable the visual detection of drugs, with each drug producing a distinct and easily discernible colour.

Future research endeavours will be dedicated to improving the printed µPADs by reducing the standard deviations of channel width. Additionally, there will be a focus on conducting concentration-based studies of the mentioned drugs on printed µPADs and exploring their potential utilization for sensing biological fluids. Furthermore, efforts will be made to advance the applications of these devices in diagnostics and pathology.

## Data Availability

The reaction schematics, preliminary solution preparation table, comparison of properties of different filter papers and LOD calculation are provided (as text, figures, mathematical expression and tables) in electronic supplementary material [62]. The raw data for AFM analysis, UV–VIS spectroscopy along with the deconvolution analysis of plots in Origin software are uploaded at Dryad Digital Repository: https://doi.org/10.5061/dryad.79cnp5j1v [61].
